# Common Endothelial Nitric Oxide Synthase Single Nucleotide Polymorphisms are not Related With the Risk for Restless Legs Syndrome

**DOI:** 10.3389/fphar.2021.618989

**Published:** 2021-02-25

**Authors:** Félix Javier Jiménez-Jiménez, Blanca G. Agúndez, Javier Gómez-Tabales, Hortensia Alonso-Navarro, Laura Turpín-Fenoll, Jorge Millán-Pascual, Mónica Díez-Fairén, Ignacio Álvarez, Pau Pastor, Marisol Calleja, Rafael García-Ruiz, Santiago Navarro-Muñoz, Marta Recio-Bermejo, José Francisco Plaza-Nieto, Esteban García-Albea, Elena García-Martín, José A. G. Agúndez

**Affiliations:** ^1^ Section of Neurology, Hospital Universitario del Sureste, Arganda del Rey, Spain; ^2^ UNEx, ARADyAL, University Institute of Molecular Pathology Biomarkers, Cáceres, Spain; ^3^ Section of Neurology, Hospital La Mancha-Centro, Alcázar de San Juan, Spain; ^4^ Fundació per la Recerca Biomèdica i Social Mútua de Terrassa, Barcelona, Spain; ^5^ Movement Disorders Unit, Department of Neurology, Hospital Universitari Mutua de Terrassa, Barcelona, Spain; ^6^ Department of Medicine-Neurology, Universidad de Alcalá, Alcalá de Henares, Spain

**Keywords:** restless legs syndrome, genetics, genetic polymorphisms, nitric oxide, nitric oxide synthase genes, risk factors

## Abstract

Because nitric oxide and endothelial dysfunction could play a role in the pathogenesis of idiopathic restless legs syndrome (RLS), as was suggested by some preliminary data, we investigated the possible association between the rs2070744 variants in the *endothelial nitric oxide synthase (eNOS* or *NOS3)* gene (chromosome 7q36.1) and the risk for RLS in a Caucasian Spanish population. We assessed the frequencies of *NOS3* single nucleotide polymorphisms (SNPs) rs2070744, rs1799983, and rs79467411 genotypes and allelic variants in 273 patients with idiopathic RLS and 325 healthy controls using a *TaqMan*-based qPCR assay. We also analyzed the possible influence of genotype frequency on age at onset of RLS symptoms, gender, family history of RLS, and response to drugs commonly used in the treatment of RLS such as dopaminergic drugs, clonazepam, and GABAergic drugs. The frequencies of genotypes and allelic variants were not associated with the risk for RLS and were not influenced by gender, age, and positive family history of RLS. We identified weak statistical associations of the SNP rs1799983 with the response to dopamine agonists (Pc = 0.018 for the rs1799983 G/T genotype) and of the SNP rs79467411 with the response to clonazepam (Pc = 0.018 for the rs79467411 G allele), although these findings should be cautiously interpreted and require further confirmation. These associations aside, our findings suggest that common *NOS3* SNPs are not associated with the risk for idiopathic RLS in Caucasian Spanish people.

## Introduction

Restless legs syndrome (RLS) or Willis-Ekbom disease (WED) is a high prevalence neurological disorder (Koo, 2015) mainly characterized by sensorimotor symptoms, with well-established diagnostic criteria (Allen et al., 2014). Despite the causative genes of RLS have not been definitively identified, initial Genomic Wide Association Studies (GWAS) identified six susceptibility genes, and a further meta-analysis of GWAS identified 13 new susceptibility loci and confirmed these associations (Schormair et al., 2017; Jiménez-Jiménez et al., 2018a). These 19 susceptibility loci should explain 11.7% of RLS heritability (Schormair et al., 2017). Results of several case-control association studies on candidate genes, which need further replication, have suggested a possible contribution in modifying the risk for RLS of *vitamin D3 receptor (VDR)* rs731236 (Jiménez-Jiménez et al., 2018a; Jiménez-Jiménez et al., 2015b), *heme-oxygenase (HMOX1)* rs2071746 (García-Martín et al., 2015; Jiménez-Jiménez et al., 2018a), allele 2 of the complex microsatellite repeat Rep1 within the *alpha-synuclein* (*SNCA*) gene promoter (Jiménez-Jiménez et al., 2018a), *gamma-aminobutyric acid (GABA) receptor rho3* (*GABRR3*) rs832032 (Jiménez-Jiménez et al., 2018b), and *alcohol-dehydrogenase 1B (ADH1B)* rs1229984 (Jiménez-Jiménez et al., 2017b). A weak association between RLS risk and the *neuronal nitric oxide synthase* (*nNOS* or *NOS1*) rs7977109 variant was described in a three-stage association study in Germans (Winkelman et al., 2008), but this association was not found in a replication study involving Spanish Caucasian population (Jiménez-Jiménez et al., 2015a).

The neurochemical features of RLS are neither well known. Together with dopaminergic dysfunction and iron deficiency as the most consistent hypothesis, and the possible contribution of glutamatergic, GABAergic, and adenosinergic neurotransmission among others (Jiménez-Jiménez et al., 2019), several preliminary findings suggested a possible of nitric oxide (NO) and oxidative stress in the RLS pathophysiology:

A) Increased expression of NOS1 in the *substantia nigra* of patients diagnosed with idiopathic RLS was found in an immunohistochemical study (Patton et al., 2011).
B) Decreased serum levels of nitrites (markers of NO) associated with increased plasma levels of advanced oxidation protein products and serum levels of the marker of lipid peroxidation malonaldehyde (MDA), and decreased serum levels of the antioxidant molecule thiol, in 22 patients with idiopathic RLS compared with 20 controls (Baskol et al., 2012).
C) Increased NOS expression in the spinal cord (thoracic intermediolateral nucleus) in *DRD3 knock-out* mice, compared with wild-type mice (Clemens et al., 2005).

Biological functions of NO include the maintaining of the arterial vasodilatory tone, the inhibition of platelet aggregation, antitumor and antimicrobial activities, mediation of macrophage cytotoxicity, and actions on neurotransmission processes (Molina et al., 1998). NO, depending on its redox form, can have neuroprotective effects through N-methyl-D-aspartate (NMDA) glutamatergic receptor blockade, or act as a reactive free radical (Molina et al., 1998). NO is synthesized from L-arginine by the action of the three isoforms of NOS: neuronal NOS (nNOS or NOS1), inducible NOS (iNOS or NOS2), and endothelial NOS (eNOS or NOS3). The protein eNOS is encoded by the *NOS3* or *eNOS* gene (chromosome 7q36.1; Gene Identity 4846, MIM 163729) (Link http://www.ncbi.nlm.nih.gov/gene/4846).

Several studies have described a possible role of vascular factors in the pathophysiology of RLS, mainly increased arterial stiffness (Han et al., 2019), impaired cerebral and peripheral vascular endothelial dysfunction (Koh et al., 2015; Kim et al., 2019), and increased capillary network in the skeletal muscle (Larsson et al., 2007; Wåhlin-Larsson et al., 2009), the later related with vascular endothelial growth factor (VEGF) (Wåhlin-Larsson et al., 2009). In contrast, recent studies have shown decreased serum levels of endocan (a marker of endothelial dysfunction) (Celik et al., 2015), and lectin-like oxidized Low-Density Lipoprotein Receptor-1 (LOX-1, a proatherogenic substance that is expressed in endothelial cells under proatherogenic conditions) (Halac et al., 2016) in patients diagnosed with iRLS compared with control groups, suggesting that iRLS patients show decreased risk for atherosclerosis and endothelial dysfunction.

Because decreased NOS3 expression is related to endothelial dysfunction (Napoli and Ignarro, 2001; Napoli et al., 2006), we investigated the possible association between the rs2070744, rs 1799983, and rs79467411 single nucleotide polymorphisms (SNPs) in the *NOS3* gene and the risk for RLS in Caucasian Spanish people. As a secondary analysis, we studied the possible influence of these SNPs in age at onset and severity of RLS, gender, positive family history of RLS, and the response of RLS symptoms to several treatments.

## Patients and Methods

### Patients and Controls

This study involved 273 patients diagnosed with idiopathic RLS according to the International Restless Legs Syndrome Study Group (IRLSSG) diagnostic criteria (Allen et al., 2014), after exclusion of secondary causes as was described elsewhere (Jiménez-Jiménez et al., 2017b), and 325 age- and sex-matched healthy controls. Table 1 summarizes the demographic and clinical data of both groups. RLS patients were recruited from the Movement Disorders Unit of 4 hospitals, and healthy controls (none of them have personal or familial history of RLS, tremor, or other movement disorders) were staff or students from the University of Extremadura. Sixty percent of RLS patients were involved in other case-control genetic association studies published by our group (Jiménez-Jiménez et al., 2013; Roco et al., 2013; Jiménez-Jiménez et al., 2014; García-Martín et al., 2015; Jiménez-Jiménez et al., 2015a; Jiménez-Jiménez et al., 2015b; Jiménez-Jiménez et al., 2017a; Jiménez-Jiménez et al., 2017b; Jiménez-Jiménez et al., 2018b).

**TABLE 1:** Demographic and clinical data of the series studied.

Group	RLS patients (n = 273)	Healthy controls (n = 325)
Age (years): Mean (SD); range	55.9 (14.9); 21–94	51.7 (16.4); 19–96
Age at onset (years): Mean (SD); range	43.6 (17.9); 5–82	NA
Age at onset <15 years: N (%); range	157 (55.5); 2–15	NA
Female N (%)	208 (76.2)	247 (76.0)
Positive family history: N (%)	181 (66.3)	NA
IRLSSG scale score, mean (SD)	24.7 (8.1)	NA

### Genotyping of NOS3 Single Nucleotide Polymorphisms

Genomic DNA, obtained from venous blood samples of participants in the study, was used for genotyping, which was performed by using pre-designed specific TaqMan probes for the SNPs rs1799983 (Asp298Glu; C___3219460_20), rs79467411 (Cys602Tyr; C_100840566_10) and the intronic rs2070744 SNP (C__15903863_10), all by Life Technologies, Alcobendas, Madrid, Spain). All the SNPs were analyzed by triplicate in a QuantStudio 3 thermocycler (Life Technologies, Alcobendas, Madrid, Spain). Full details of the genotyping procedure, which was identical for all SNPs, are described elsewhere (Jiménez-Jiménez et al., 2015a). The promoter rs2070744 SNP was included in the study because it has several clinical associations and it is related to increased mRNA expression (Kittel-Schneider et al., 2015) The two missense *NOS3* SNPs were selected according to their allele frequencies in public databases such as the Genome Aggregation Database (gnomAD; https://gnomad.broadinstitute.org/), because both displayed minor allele frequencies higher than, or around, 10%, which is adequate to reach a high statistical power.

### Statistical Analysis

The SPSS 15.0 version for Windows (SPSS Inc., Chicago, Illinois, United States) was used to perform statistical analysis. Confirmation of the Hardy-Weinberg equilibrium, both in RLS patients and controls, was done with the online program https://ihg.gsf.de/cgi-bin/hw/hwa1.pl. Intergroup comparison values (both between the whole series of RLS patients and controls, between RLS patients and controls considering each gender separately and between RLS patients depending on the presence or absence of a family history of RLS or the response of RLS symptoms to the therapy) were done using the chi-square test (or the Fisher’s exact test where appropriate). The 95% CIs and the negative predictive values were also calculated (Altman and Bland, 1994). False Discovery Rate (FDR) correction was used for multiple comparison adjustments (Benjamini et al., 2001).

Determination of the sample size was done from the allele frequencies observed for healthy individuals by using a genetic model analyzing the minor allele frequency with an odds ratio (OR) value = 1.5 (*α* = 0.05). According to the sample size of this study, the statistical power (two-tailed association) for variant alleles, was as follows: rs2070744 = 93.7%, rs1799983 = 93.1%, and rs79467411 = 86.5%.

The comparisons of mean age at onset of RLS symptoms and severity of RLS symptoms according to the IRLSSG scale (Walters et al., 2003) between genotypes were done by using a *t*-test for independent samples.

## Results

Hardy-Weinberg’s equilibrium for all genotypes and allelic variants frequencies was present both in RLS and healthy control groups. The frequencies of genotypes and allelic variants did not differ significantly between RLS patients and controls, neither in the whole series (Table 2), or analyzing men and females separately (Table 3), and were not influenced by the positivity of family history of RLS (Table 4). With regard to drug response, we analyzed the genotypes stratifying patients according to their response to dopaminergic drugs, clonazepam, and GABAergic drugs (Table 5). We identified a statistically significant increased frequency of patients with the rs1799983 (G/T) genotype in patients not responding to dopamine agonists (DAs), as compared with patients who responded to these drugs (*p* = 0.002). The statistical significance remained after FDR correction (Pc = 0.018). However, this association is likely to be due to chance, since the subgroup of patients not responding to DAs is small, and no increased frequency of homozygous rs1799983 (G/G) patients was observed among patients not responding to DAs. Also, we identified a low frequency of patients not responding to clonazepam and carrying the rs79467411 (G/G) genotype (*p* = 0.007), although such statistical significance became marginal after FDR correction (Pc = 0.063). The rs79467411 allele frequencies were also different among patients responding or not responding to clonazepam (*p* = 0.006; Table 5). After FRD adjustment the *p*-value remained significant (Pc = 0.018). These findings, however, should be interpreted cautiously since the number of patients not responding to clonazepam was very small (n = 16). Age at onset of RLS (Table 6), and RLS severity (Table 7), did not differ significantly between the different genotypes in RLS patients.

**TABLE 2:** *NOS3* genotypes and allelic variants of patients with RLS and healthy volunteers.

	RLS patients (N = 273, 546 alleles)	Controls (N = 325, 650 alleles)	Intergroup comparison Or (95% CI), *p*; NPV (95% CI)
Genotypes			
rs1799983 (T/T)	36 (13.2; 9.2–17.2)	47 (14.5; 10.6–18.3)	0.90 (0.56–1.43); 0.654; 0.54 (0.52–0.56)
rs1799983 (G/T)	128 (46.9; 41.0–52.8)	149 (45.8; 40.4–51.3)	1.04 (0.76–1.44); 0.800; 0.55 (0.51–0.59)
rs1799983 (G/G)	109 (39.9; 34.1–45.7)	129 (39.7; 34.4–45.0)	1.01 (0.73–1.40); 0.954; 0.54 (0.51–0.58)
rs79467411 (G/G)	160 (58.6; 52.8–64.5)	190 (58.5; 53.1–63.8)	1.01 (0.73–1.40), 0.971; 0.54 (0.50–0.59)
rs79467411 (G/A)	100 (36.6; 30.9–42.3)	124 (38.2; 32.9–43.4)	0.94 (0.67–1.31); 0.702; 0.54 (0.51–0.57)
rs79467411 (A/A)	13 (4.8; 2.2–7.3)	11 (3.4; 1.4–5.4)	1.43 (0.63–3.24); 0.393; 0.55 (0.54–0.56)
rs2070744 (T/T)	76 (27.8; 22.5–33.2)	101 (31.1; 26.0–36.1)	0.86 (0.60–1.22); 0.388; 0.53 (0.51–0.56)
rs2070744 (T/C)	135 (49.5; 43.5–55.4)	159 (48.9; 43.5–54.4)	1.02 (0.74–1.41); 0.898; 0.55 (0.51–0.59)
rs2070744 (C/C)	62 (22.7; 17.7–27.7)	65 (20.0; 15.7–24.3)	1.18 (0.79–1.74); 0.420; 0.55 (0.53–0.57)
Alleles			
rs1799983 (T)	200 (36.6; 32.6–40.7)	243 (37.4; 33.7–41.1)	0.97 (0.77–1.23); 0.788; 0.54 (0.52–0.56)
rs1799983 (G)	346 (63.4; 59.3–67.4)	407 (62.6; 58.9–66.3)	1.03 (0.82–1.31); 0.788; 0.55 (0.51–0.59)
rs79467411 (G)	420 (76.9; 73.4–80.5)	504 (77.5; 74.3–80.7)	0.97 (0.74–1.27); 0.800; 0.54 (0.48–0.59)
rs79467411 (A)	126 (23.1; 19.5–26.6)	146 (22.5; 19.3–25.7)	1.04 (0.79–1.36); 0.800; 0.55 (0.53–0.56)
rs2070744 (T)	287 (52.6; 48.4–56.8)	361 (55.5; 51.7–59.4)	0.89 (0.71–1.12); 0.304; 0.53 (0.50–0.56)
rs2070744 (C)	259 (47.4; 43.2–51.6)	289 (44.5; 40.6–48.3)	1.13 (0.90–1.42); 0.304; 0.56 (0.53–0.58)

The values in each cell represent: number (percentage; 95% CIs).

**TABLE 3:** *NOS3* genotypes and allelic variants of patients with RLS and healthy volunteers distributed by gender.

	RLS women (N = 208, 416 alleles)	Control women (N = 247, 494 alleles)	Intergroup comparisoN values OR (95%CI) *p*	NPV (95%CI)	RLS men (N = 65, 130 alleles)	Control men (N = 78, 156 alleles)	Intergroup comparison values OR (95%CI) p	NPV (95%CI)
Genotypes								
rs1799983 (T/T)	28 (13.5; 8.8–18.1)	36 (14.6; 10.2–19.0)	0.91 (0.54–1.55); 0.734	0.54 (0.52–0.56)	8 (12.3; 4.3–20.3)	11 (14.1; 6.4–21.8)	0.86 (0.32–2.27); 0.754	0.54 (0.51–0.58)
rs1799983 (G/T)	91 (43.8; 37.0–50.5)	113 (45.7; 39.5–52.0)	0.92 (0.64–1.34); 0.670	0.53 (0.49–0.58)	37 (56.9; 44.9–69.0)	36 (46.2; 35.1–57.2)	1.54 (0.80–2.99); 0.201	0.60 (0.51–0.69)
rs1799983 (G/G)	89 (42.8; 36.1–49.5)	98 (39.7; 33.6–45.8)	1.14 (0.78–1.65); 0.502	0.56 (0.52–0.60)	20 (30.8; 19.5–42.0)	31 (39.7; 28.9–50.6)	0.67 (0.34–1.35); 0.266	0.51 (0.45–0.58)
rs79467411 (G/G)	122 (58.7; 52.0–65.3)	145 (58.7; 52.6–64.8)	1.00 (0.69–1.45); 0.991	0.54 (0.49–0.60)	38 (58.5; 46.5–70.4)	45 (57.7; 46.7–68.7)	1.03 (0.53–2.01); 0.926	0.55 (0.45–0.65)
rs79467411 (G/A)	74 (35.6; 29.1–42.1)	94 (38.1; 32.0–44.1)	0.90 (0.61–1.32); 0.585	0.53 (0.50–0.57)	26 (40.0; 28.1–51.9)	30 (38.5; 27.7–49.3)	1.07 (0.54–2.09); 0.852	0.55 (0.48–0.62)
rs79467411 (A/A)	12 (5.8; 2.6–8.9)	8 (3.2; 1.0–5.4)	1.83 (0.73–4.56); 0.190	0.55 (0.54–0.56)	1 (1.5; –1.5–4.5)	3 (3.8; –0.4–8.1)	0.39 (0.04–3.85); 0.406	0.54 (0.53–0.56)
rs2070744 (T/T)	60 (28.8; 22.7–35.0)	77 (31.2; 25.4–37.0)	0.90 (0.60–1.34); 0.590	0.54 (0.50–0.57)	16 (24.6; 14.1–35.1)	24 (30.8; 20.5–41.0)	0.74 (0.35–1.54); 0.416	0.52 (0.47–0.58)
rs2070744 (T/C)	105 (50.5; 43.7–57.3)	121 (49.0; 42.8–55.2)	1.06 (0.73–1.54); 0.751	0.55 (0.50–0.60)	30 (46.2; 34.0–58.3)	38 (48.7; 37.6–59.8)	0.90 (0.47–1.75); 0.761	0.53 (0.45–0.62)
rs2070744 (C/C)	43 (20.7; 15.2–26.2)	49 (19.8; 14.9–24.8)	1.05 (0.67–1.67); 0.825	0.55 (0.52–0.57)	19 (29.2; 18.2–40.3)	16 (20.5; 11.6–29.5)	1.60 (0.74–3.45); 0.229	0.57 (0.52–0.62)
Alleles								
rs1799983 (T)	147 (35.3; 30.7–39.9)	185 (37.4; 33.2–41.7)	0.90 (0.70–1.20); 0.510	0.54 (0.51–0.56)	53 (40.8; 32.3–49.2)	58 (37.2; 29.6–44.8)	1.16 (0.72–1.87); 0.536	0.56 (0.51–0.61)
rs1799983 (G)	269 (64.7; 60.1–69.3)	309 (62.6; 58.3–66.8)	1.10 (0.84–1.44); 0.510	0.56 (0.51–0.60)	77 (59.2; 50.8–67.7)	98 (62.8; 55.2–70.4)	0.86 (0.53–1.39); 0.536	0.52 (0.45–0.60)
rs79467411 (G)	318 (76.4; 72.4–80.5)	384 (77.7; 74.1–81.4)	0.93 (0.68–1.27); 0.644	0.53 (0.47–0.59)	102 (78.5; 71.4–85.5)	120 (76.9; 70.3–83.5)	1.09 (0.62–1.91); 0.756	0.56 (0.45–0.67)
rs79467411 (A)	98 (23.6; 19.5–27.6)	110 (22.3; 18.6–25.9)	1.08 (0.79–1.47); 0.644	0.55 (0.53–0.67)	28 (21.5; 14.5–28.6)	36 (23.1; 16.5–29.7)	0.92 (0.52–1.60); 0.756	0.54 (0.51–0.57)
rs2070744 (T)	225 (54.1; 49.3–58.9)	275 (55.7; 51.3–60.0)	0.94 (0.72–1.22); 0.633	0.53 (0.50–0.57)	62 (47.7; 39.1–56.3)	86 (55.1; 47.3–62.9)	0.74 (0.47–1.18); 0.211	0.51 (0.45–0.57)
rs2070744 (C)	191 (45.9; 41.1–50.7)	219 (44.3; 40.0–48.7)	1.07 (0.82–1.39); 0.633	0.55 (0.52–0.58)	68 (52.3; 43.7–60.9)	70 (44.9; 37.1–52.7)	1.35 (0.85–2.15); 0.211	0.58 (0.52–0.64)

The values in each cell represent: number (percentage; 95% CIs).

**TABLE 4:** *NOS3* genotypes and allelic variants of patients with RLS distributed by family history.

Genotype	Positive family history of RLS (N = 181, 362 alleles)	Negative family history of RLS (N = 92, 184 alleles)	Intergroup comparison values OR (95% CI) *p*; NPV OR (95% CI)
Genotypes			
rs1799983 (T/T)	23 (12.7; 7.9–17.6)	13 (14.1; 7.0–21.2)	0.89 (0.43–1.84); 0.743; 0.33 (0.31–0.36)
rs1799983 (G/T)	88 (48.6; 41.3–55.9)	40 (43.5; 33.3–53.6)	1.23 (0.74–2.04); 0.422; 0.36 (0.30–0.41)
rs1799983 (G/G)	70 (38.7; 31.6–45.8)	39 (42.4; 32.3–52.5)	0.86 (0.51–1.43); 0.554; 0.32 (0.27–0.37)
rs79467411 (G/G)	106 (58.6; 51.4–65.7)	54 (58.7; 48.6–68.8)	1.00 (0.60–1.66); 0.983; 0.34 (0.27–0.41)
rs79467411 (G/A)	66 (36.5; 29.5–43.5)	34 (37.0; 27.1–46.8)	0.98 (0.58–1.65); 0.936; 0.34 (0.29–0.38)
rs79467411 (A/A)	9 (5.0; 1.8–8.1)	4 (4.3; 0.2–8.5)	1.15 (0.35–3.84); 0.819; 0.34 (0.32–0.35)
rs2070744 (T/T)	53 (29.3; 22.7–35.9)	23 (25.0; 16.2–33.8)	1.24 (0.70–2.20); 0.456; 0.35 (0.31–0.38)
rs2070744 (T/C)	92 (50.8; 43.5–58.1)	43 (46.7; 36.5–56.9)	1.18 (0.71–1.95); 0.524; 0.36 (0.30–0.41)
rs2070744 (C/C)	36 (19.9; 14.1–25.7)	26 (28.3; 19.1–37.5)	0.63 (0.35–1.13); 0.119; 0.31 (0.28–0.35)
Alleles			
rs1799983 (T)	134 (37.0; 32.0–42.0)	66 (35.9; 28.9–42.8)	1.05 (0.73–1.52); 0.793; 0.34 (0.31–0.37)
rs1799983 (G)	228 (63.0; 58.0–68.0)	118 (64.1; 57.2–71.1)	0.95 (0.66–1.38); 0.793; 0.33 (0.28–0.39)
rs79467411 (G)	278 (76.8; 72.4–81.1)	142 (77.2; 71.1–83.2)	0.98 (0.64–1.49); 0.921; 0.33 (0.26–0.41)
rs79467411 (A)	84 (23.2; 18.9–27.6)	42 (22.8; 16.8–28.9)	1.02 (0.67–1.56); 0.921; 0.34 (0.32–0.36)
rs2070744 (T)	198 (54.7; 49.6–59.8)	89 (48.4; 41.1–55.6)	1.29 (0.90–1.84); 0.162; 0.38 (0.32–0.41)
rs2070744 (C)	164 (45.3; 40.2–50.4)	95 (51.6; 44.4–58.9)	0.78 (0.54–1.12); 0.162; 0.31 (0.27–0.35)

The values in each cell represent number (percentage; 95% CIs). Crude p values are shown. NPV: negative predictive value.

**TABLE 5:** *NOS3* genotypes and allelic variants of patients with RLS distributed by response to dopamine agonists, clonazepam, and GABAergic drugs.

	Positive response to dopamine agonists (N = 211, 422 alleles)	Negative response to dopamine agonists (N = 18, 36 alleles)	Intergroup comparison values OR (95%CI) *p*	Positive Response CNZ (N = 88, 176 alleles)	Negative Response CNZ (N = 16, 32 alleles)	Intergroup comparison values OR (95%CI) *p*	Positive response GABA (N = 50, 100 alleles)	Negative response GABA (N = 11, 22 alleles)	Intergroup comparison values OR (95%CI) *p*
Genotypes									
rs1799983 (T/T)	29 (13.7; 9.1–18.4)	1 (5.6; −5.0–16.1)	2.71 (0.35–21.14); 0.324	13 (14.8; 7.4–22.2)	0 (0.0; 0.0–0.0)	1.21^<u>a</u>^ (0.87–1.21); 0.102	6 (12.0; 3.0–21.0)	1 (9.1; −7.9–26.1)	1.36 (0.15–12.63); 0.786
rs1799983 (G/T)	95 (45.0; 38.3–51.7)	14 (83.3; 66.1–100.6)	0.16 (0.05–0.58); 0.002	39 (44.3; 33.9–54.7)	8 (50.0; 25.5–74.5)	0.80 (0.27–2.31); 0.676	24 (48.0; 34.2–61.8)	7 (63.6; 35.2–92.1)	0.53 (0.14–2.03); 0.352
rs1799983 (G/G)	87 (41.2; 34.6–47.9)	2 (11.1; −3.4–25.6)	5.26 (1.17–23.60); 0.017	36 (40.9; 30.6–51.2)	8 (50.0; 25.5–74.5)	0.67 (0.24–2.02); 0.500	20 (40.0; 26.4–53.6)	3 (27.3; 1.0–53.6)	1.78 (0.42–7.52); 0.434
rs79467411 (G/G)	121 (57.3; 50.7–64.0)	10 (55.6; 32.6–78.5)	1.08 (0.41–2.83); 0.883	59 (67.0; 57.2–76.9)	5 (31.3; 8.5–54.0)	4.47 (1.42–14.1); 0.007	33 (66.0; 52.9–79.1)	7 (63.6; 35.2–92.1)	1.12 (0.28–4.33); 0.882
rs79467411 (G/A)	80 (37.9; 31.4–44.5)	6 (33.3; 11.6–55.1)	1.22 (0.44–3.38); 0.701	29 (33.0; 23.1–42.8)	10 (62.5; 38.8–86.2)	0.30 (0.10–0.89); 0.025	15 (30.0; 17.3–42.7)	4 (36.4; 7.9–64.8)	0.75 (0.19–2.95); 0.682
rs79467411 (A/A)	10 (4.7; 1.9–7.6)	2 (11.1; −3.4–25.6)	0.40 (0.08–1.97); 0.245	0 (0.0; 0.0–0.0)	1 (6.3; −5.6–18.1)	0.00^<u>a</u>^ (0.00–1.12); 0.019	2 (4.0; −1.4–9.4)	0 (0.0; 0.0–0.0)	1.23^<u>a</u>^ (0.24–1.23); 0.504
rs2070744 (T/T)	57 (27.0; 21.0–33.0)	4 (22.2; 3.0–41.4)	1.30 (0.41–4.10); 0.660	28 (31.8; 22.1–41.5)	7 (43.8; 19.4–68.1)	0.60 (0.20–1.78); 0.355	14 (28.0; 15.6–40.4)	1 (9.1; −7.9–26.1)	3.89 (0.46–33.26); 0.191
rs2070744 (T/C)	100 (47.4; 40.7–54.1)	12 (66.7; 44.9–88.4)	0.45 (0.16–1.25); 0.117	40 (45.5; 35.1–55.9)	7 (43.8; 19.4–68.1)	1.07 (0.37–3.31); 0.900	27 (54.0; 40.2–67.8)	6 (54.5; 25.1–84.0)	0.99 (0.26–3.63); 0.974
rs2070744 (C/C)	54 (25.6; 19.7–31.5)	2 (11.1; −3.4–25.6)	2.57 (0.61–12.36); 0.171	20 (22.7; 14.0–31.5)	2 (12.5; −3.7–28.7)	2.06 (0.43–9.83); 0.359	9 (18.0; 7.4–28.6)	4 (36.4; 7.9–64.8)	0.39 (0.09–1.60); 0.182
Alleles									
rs1799983 (T)	153 (36.3; 31.7–40.8)	17 (47.2; 30.9–63.5)	0.64 (0.321.26); 0.192	65 (36.9; 29.8–44.1)	8 (25.0; 10.0–40.0)	1.76 (0.75–4.14); 0.194	36 (36.0; 26.6–45.4)	9 (40.9; 20.4–61.5)	0.81 (0.32–2.09); 0.667
rs1799983 (G)	269 (63.7; 59.2–68.3)	19 (52.8; 36.5–69.1)	1.57 (0.79–3.12); 0.192	111 (63.1; 55.9–70.2)	24 (75.0; 60.0–90.0)	0.57 (0.24–1.34); 0.194	64 (64.0; 54.6–73.4)	13 (59.1; 38.5–79.6)	1.23 (0.48–3.16); 0.667
rs79467411 (G)	322 (76.3; 72.2–80.4)	26 (72.2; 57.6–86.9)	1.24 (0.58–2.66); 0.583	147 (83.5; 78.0–89.0)	20 (62.5; 45.7–79.3)	3.04 (1.34–6.90); 0.006	81 (81.0; 73.3–88.7)	18 (81.8; 65.7–97.9)	0.95 (0.29–3.12); 0.930
rs79467411 (A)	100 (23.7; 19.6–27.8)	10 (27.8; 13.1–42.4)	0.81 (0.38–1.73); 0.583	29 (16.5; 11.0–22.0)	12 (37.5; 20.7–54.3)	0.33 (0.15–0.75); 0.006	19 (19.0; 11.3–26.7)	4 (18.2; 2.1–34.3)	1.06 (0.32–3.48); 0.930
rs2070744 (T)	214 (50.7; 45.9–55.5)	20 (55.6; 39.3–71.8)	0.82 (0.42–1.63); 0.577	96 (54.5; 47.2–61.9)	21 (65.6; 49.2–82.1)	0.63 (0.29–1.38); 0.246	55 (55.0; 45.2–64.8)	8 (36.4; 16.3–56.5)	2.14 (0.82–5.55); 0.115
rs2070744 (C)	208 (49.3; 44.5–54.1)	16 (44.4; 28.2–60.7)	1.22 (0.61–2.41); 0.577	80 (45.5; 38.1–52.8)	11 (34.4; 17.9–50.8)	1.59 (0.72–3.50), 0.246	45 (45.0; 35.2–54.8)	14 (63.6; 43.5–83.7)	0.47 (0.18–1.21); 0.115

The values in each cell represent number (percentage; 95% CIs).

^a^ The Relative risk is shown instead of the Odds Ratio, because one of the values is equal to 0.

**TABLE 6:** Age at onset of RLS according the *NOS3* genotypes.

	Age at onset (years)	Two tailed *t*-Test compared to non-mutated	Two tailed *t*-Test compared to heterozygous
rs1799983 (T/T)	45.6 ± 15.8		
rs1799983 (G/T)	43.7 ± 18.3	0.572	
rs1799983 (G/G)	43.0 ± 17.8	0.439	0.773
rs79467411 (G/G)	43.1 ± 17.4		
rs79467411 (G/A)	44.1 ± 18.1	0.666	
rs79467411 (A/A)	47.2 ± 19.7	0.412	0.557
rs2070744 (T/T)	45.9 ± 16.5		
rs2070744 (T/C)	42.7 ± 17.8	0.206	
rs2070744 (C/C)	42.9 ± 18.9	0.335	0.931

**TABLE 7:** IRLSSGRS according to *NOS3* genotypes.

	Irlssgrs	Two tailed *t*-Test compared to non-mutated	Two tailed *t*-Test compared to heterozygous
rs1799983 (T/T)	23.0 ± 4.8		
rs1799983 (G/T)	24.5 ± 7.0	0.230	
rs1799983 (G/G)	25.0 ± 6.8	0.120	0.633
rs79467411 (G/G)	25.0 ± 6.2		
rs79467411 (G/A)	24.1 ± 6.5	0.265	
rs79467411 (A/A)	25.6 ± 6.9	0.746	0.459
rs2070744 (T/T)	23.7 ± 6.7		
rs2070744 (T/C)	24.8 ± 7.0	0.240	
rs2070744 (C/C)	24.9 ± 6.0	0.275	0.959

## Discussion

The possible role of *NOS3* variants on the risk for neurological diseases has been the matter of several recent reports. In this regard, it has been reported an increased risk for delayed cerebral ischemia following aneurismal subarachnoid hemorrhage (Hendrix et al., 2017) and hypoxic-ischemic encephalopathy (Wu et al., 2016). One study in the Iranian population showed an association between *NOS3* rs2070744 and multiple sclerosis (Heidari et al., 2017), but this association was not replicated in other populations (AlFadhli et al., 2013; Agúndez et al., 2020). It has been described increased risk for stroke in a population-based study in the United States (Fan et al., 2010), but this association was not replicated by a case-control association study in Turkey (Anlıaçık et al., 2019). A meta-analysis showed an association between *NOS3* rs2070744CC genotype and the risk for migraine in Caucasians (Dong et al., 2018), but a replication study in the Caucasian Spanish population did not confirm this finding (García-Martín et al., 2020).

Despite the possibility that NO could play a in the pathogenesis of RLS suggested by several previous mentioned studies (Clemens et al., 2005; Patton et al., 2011; Baskol et al., 2012), the possible contribution of polymorphisms in the *NOS* genes to the risk of developing RLS has not been established. While a previously mentioned *NOS1* variant was associated with RLS risk in a study (Winkelmann et al., 2008), this was not confirmed in another one (Jiménez-Jiménez et al., 2015a).

In the present study, involving Caucasian Spanish people, we analyzed three common *NOS3* SNPs: the promoter SNP rs270744, which alters mRNA expression (Kittel-Schneider et al., 2015), as well as two common nonsynonymous SNPs: rs1799983 that induces the amino acid exchange Asp298Glu, and has clinical implications (Malik et al., 2018), and rs79467411 that induces the amino acid exchange Cys602Tyr. Other *NOS3* functional SNPs, such as rs143125350 (Arg613Gly) or rs3918166 (Arg112Gln) show extremely low minor allele frequencies (0.000 and 0.002, respectively) in Southern European subjects according to public databases (gnomAD; https://gnomad.broadinstitute.org/) and therefore were not included in the analyses. The 27-bp VNTR 4a/4b *NOS3* gene variation (Zhang et al., 2008) was not analyzed because of DNA shortage. However, it should be taken into consideration that the functional effect of the 27-bp VNTR has been determined in a very small number of individuals and quantitatively is relatively small (Zhang et al., 2008).

We did not identify any major association between common *NOS3* SNPs with the risk for RLS. Moreover, these *NOS3* variants were not related to the age at onset or with the severity of RLS assessed by the IRLSSG scale scores (Walters et al., 2003), or family history or RLS. Regarding the response to drugs usually used for the treatment of RLS, we identified weak associations that should be interpreted cautiously, and require further confirmation.

The main limitation of the present study is the relatively low sample size of the two analyzed cohorts (RLS patients and controls). While the sample size of our cohorts should be adequate to detect ORs of 1.5, it is likely that it could not be sufficient to detect more modest associations. However, taking into account this limitation, the results of this study suggest that common *NOS3* SNPs are unrelated to the risk for RLS in the Caucasian Spanish people. These results do not preclude the possibility that other SNPs in the *NOS3* gene could be associated with the modification of the risk of developing this disease.

## Data Availability

The data analyzed in this study is subject to the following licenses/restrictions: All data related to the current study, intended for reasonable use, is available from JA (University Institute of Molecular Pathology Biomarkers, University of Extremadura -UNEx ARADyAL Instituto de Salud Carlos III, Av/ de la Universidad S/N, E10071 Cáceres. Spain) and FJJ-J (Section of Neurology, Hospital del Sureste, Arganda del Rey, Madrid, Spain). Requests to access these datasets should be directed to jagundez@unex.es; fjavier.jimenez@salud.madrid.org.
